# 2725. Bacterial blood stream infections in Pediatric Liver Transplant Patients: Prevalence, Organisms and Risk factors. A Systematic review

**DOI:** 10.1093/ofid/ofad500.2336

**Published:** 2023-11-27

**Authors:** Mohamad Shieb, Rand Hasanain, Zara Arshad, Faisal A Nawaz, Rahul Kashyap, Eric J Stern

**Affiliations:** medstar georgetown university hospital, ARLINGTON, Virginia; Al Jalila Children’s Hospital, Twar, Dubai, United Arab Emirates; Shifa International Hospital, Islamabad, Islamabad, Pakistan; Department of Psychiatry, Al Amal Psychiatric Hospital, Dubai, United Arab Emirates, Dubai, Dubai, United Arab Emirates; Mayo Clinic, Rochester, Minnesota; Medstar Georgetown Pediatrics, Washington, District of Columbia

## Abstract

**Background:**

Bacterial blood stream infection (BSI) is a leading cause for mortality and morbidity in the pediatric solid organ transplant recipient population. This systematic review aims at pooling global data from the leading transplant institutions to identify the overall prevalence and risk factors associated with bacterial BSI and the most common causative organisms.

**Methods:**

A systematic review of PubMed and OVID databases was conducted for the past 20 years. Initial search from both databases had a yield of 252 unique articles which were reviewed independently by two authors. Data related to demographics, prevalence, organisms and risk factors were extracted from the articles and subsequently analysed. The systematic review was registered on PROSPERO (ID: CRD42023403206).

The PRISMA flow diagram visually summarises the screening process

**Figure:**
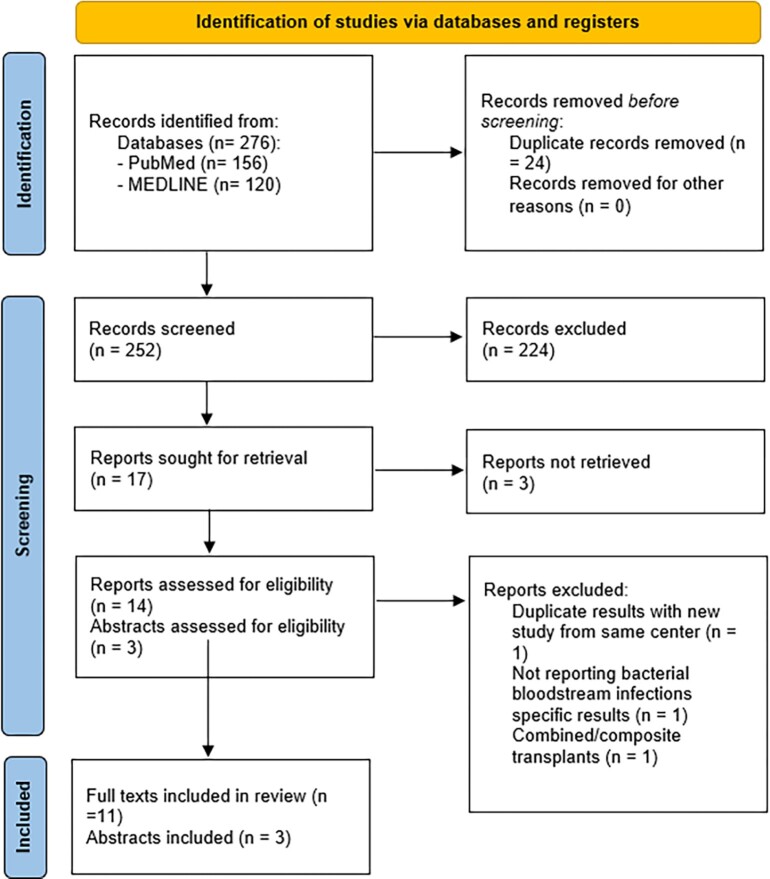

**Results:**

A total of 14 articles were included from the United States of America, France, Italy, Thailand, Japan, Spain and Korea. A total of 4215 liver transplants were included in the final analysis. The overall prevalence of BSI in pediatric liver transplant patients was 23.5% with the highest prevalence reported in Japan (39.5%) and lowest in the USA (18.7%). Mean age of the patients was 25 months and 54% were females. The most common organisms reported were Staphylococcus epidermidis at 22.8% followed by Enterococcus species (16.6%), Klebsiella species (11.9%) and Escherichia coli (11.4%). Among the risk factors studied, postoperative biliary complications, past medical history of biliary atresia and younger age were the risk factors most associated with BSI.

Prevalence of bloodstream infections (BSI) post pediatric liver transplant in included countries.

**Figure:**
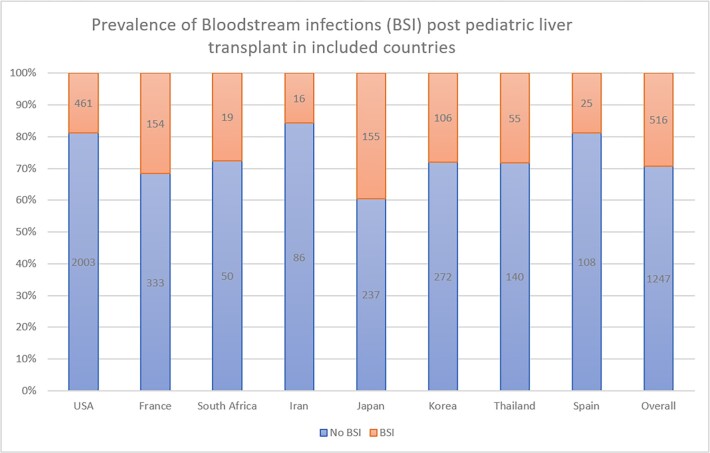

Bacterial organisms isolated in blood cultures in pediatric post liver transplant patient with a bloodstream infection.

**Figure:**
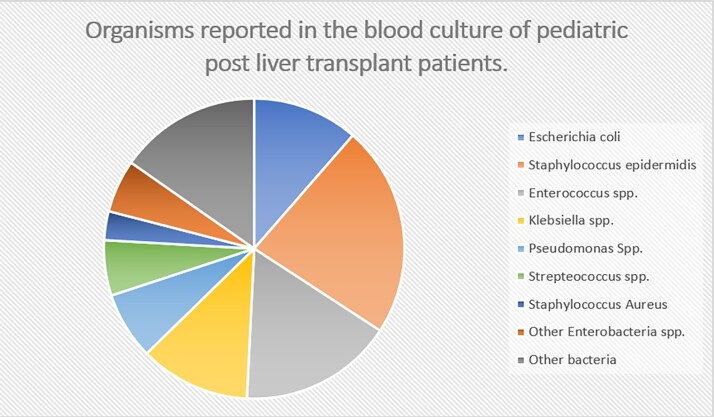

**Conclusion:**

Bacterial BSI post pediatric liver transplantation occurs with high prevalence. Further studies need to be conducted to better define risk factors for BSI and determine the best empirical antibiotic management for BSI in this population as the distribution of organisms varies greatly from otherwise healthy pediatric patients.

**Disclosures:**

**All Authors**: No reported disclosures

